# Microplastics and nanoplastics in cardiovascular disease—a narrative review with worrying links

**DOI:** 10.3389/ftox.2024.1479292

**Published:** 2024-10-10

**Authors:** Haixiang Zheng, Gianpaolo Vidili, Gavino Casu, Eliano Pio Navarese, Leonardo A. Sechi, Youren Chen

**Affiliations:** ^1^ Department of Cardiology, The Second Affiliated Hospital of Shantou University Medical College, Shantou, China; ^2^ Department of Biomedical Sciences, University of Sassari, Sassari, Italy; ^3^ Department of Medicine, Surgery, and Pharmacy, University of Sassari, Azienda Ospedaliero, Sassari, Italy; ^4^ Clinical and Experimental Cardiology, Clinical and Interventional Cardiology, University of Sassari, Sassari, Italy; ^5^ Complex Structure of Microbiology and Virology, AOU Sassari, Sassari, Italy

**Keywords:** microplastics, nanoplastics, cardiovascular disease, correlation, cardiotoxicity, environmental health

## Abstract

With the widespread use of plastic products and the increase in waste, microplastics and nanoplastics (MNPs) have become an important issue in global environmental pollution. In recent years, an increasing number of studies have shown that MNPs may have negative impacts on human health. This review aimed to explore the association between MNPs and cardiovascular disease and provide an outlook for future research. Research has shown that there may be a link between MNPs exposure and cardiovascular disease. Laboratory studies have shown that animals exposed to MNPs often exhibit abnormalities in the cardiovascular system, such as increased blood pressure, vascular inflammation, and myocardial damage. Epidemiological surveys have also revealed that people exposed to MNPs are more likely to suffer from cardiovascular diseases, such as hypertension and myocardial infarction. Although the specific impact mechanism is not fully understood, there are several possible pathways of action, including the effects of toxic substances on MNPs and interference with the endocrine system. In summary, MNPs exposure may have a negative impact on cardiovascular health, but further research is needed to confirm its specific mechanism and extent of impact to guide relevant public health and environmental policies.

## 1 Introduction

**FIGURE 1:**
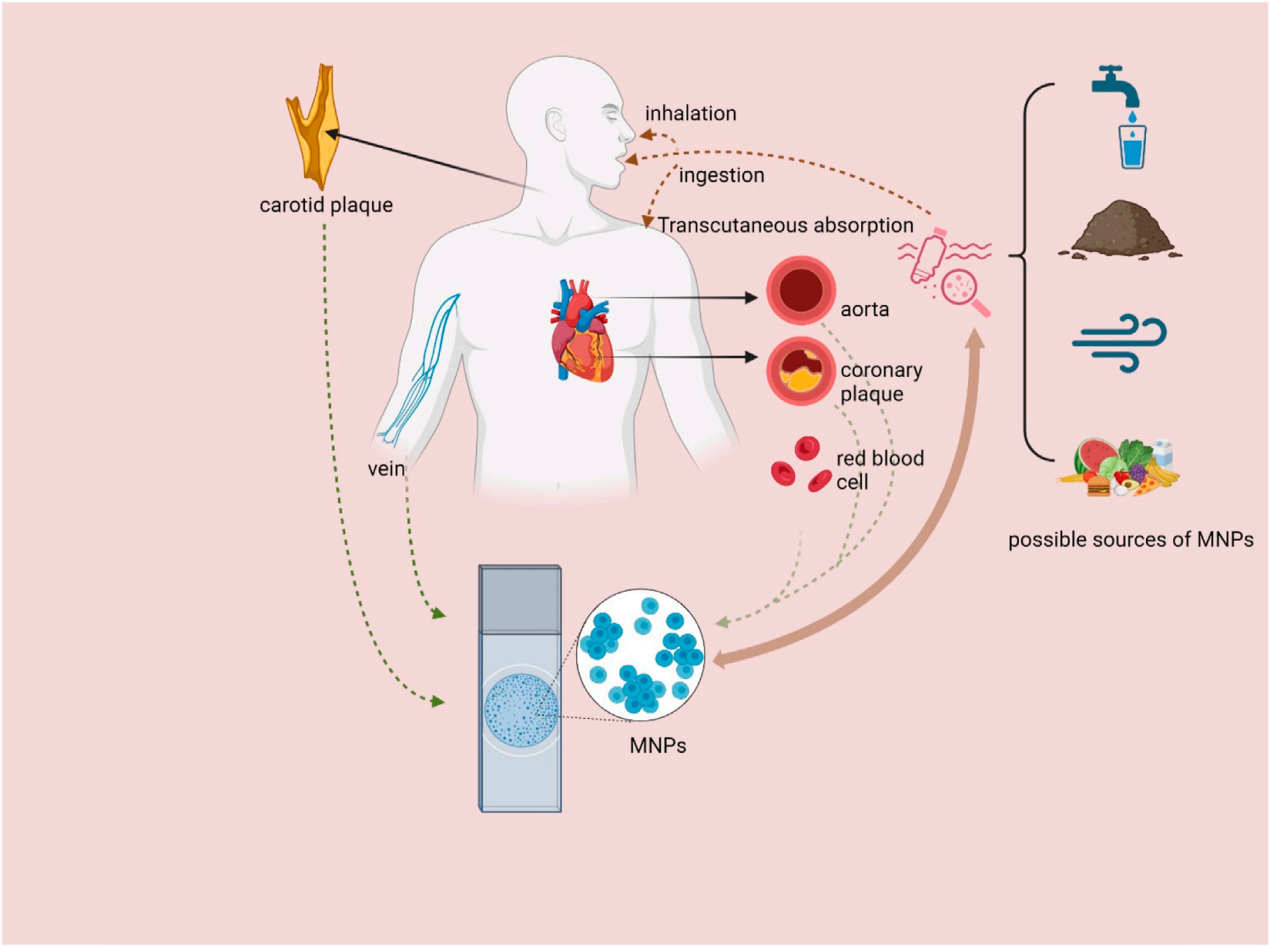
Sources of MNPs, pathways into the human body and distribution in cardiovascular-related organs and tissues. MNPs = microplastics and nanoplastics (created with BioRender.com).

Plastic products have become an indispensable part of human life in the modern world. They are widely used in food packaging, daily manufacturing, and medical equipment, among other fields. However, with the widespread use of plastic products, plastic pollution has become increasingly severe. If this trend continues, it is estimated that plastic production will continue to affect the natural environment by 2050 (Roland, 2017). Among them, microplastics and nanoplastics (MNPs) pollution, an emerging environmental pollutant, has attracted much attention (Osman et al., 2023). MNPs are plastic particles with a diameter of less than 5 mm, and their common chemical ingredients include polyethylene, polyvinyl chloride, polystyrene, and polypropylene. These contaminants originate from the wear and tear of plastic products, decomposition of plastic waste, and artificial addition of MNPs particles. MNPs are widely distributed in water, soil, air, and food, and humans are exposed to MNPs through diet, breathing, and skin contact (Figure 1). Once released into nature, MNPs trigger a series of toxicological events (Legler and Legler, 2021). MNPs also pollute the environment through ocean currents, atmospheric winds, and terrestrial phenomena, thus becoming a global environmental pollution problem (Wang et al., 2020).

With continued research on MNPs pollution, scientists have begun to discover that MNPs may have a negative impact on human health (SAPEA, 2019; Ali et al., 2024). Several studies have reported that MNPs are found in human tissues, such as the lung, liver, urine, blood, placenta, and breast milk, by noninvasive imaging methods (Table 1) (Jenner et al., 2022; Horvatits et al., 2022; Pironti et al., 2022; Leslie et al., 2022; Ragusa et al., 2021; Ragusa et al., 2022). A recent study suggested that there may be a link between MNPs exposure and cardiovascular disease (CVD), potentially through the induction of toxicity at microvascular sites, resulting in an abnormal heart rate, impaired cardiac function, pericardial effusion and myocardial fibrosis (Zhu et al., 2023). CVD, which includes hypertension, coronary heart disease, and heart failure, is a major cause of death worldwide and poses a serious threat to human health. Due to the omnipresence of MNPs in the environment, exploring the relationship between MNPs and CVD has important scientific and practical significance. The objective of this review is to provide a comprehensive overview of the current state of knowledge regarding the correlation between MNPs and CVD and to identify any gaps in the literature.

**TABLE 1:** MNPs discovered in human organ tissue.

Literature sources, Yrs	Organ tissue	Detection method	Types of MNPs	Size/Quantity/ Shape	Potential impact
Detection of microplastics in human lung tissue using muFTIR spectroscopy,2022	lung	μFTIR spectroscopy	PP, PET, resin	223.10 × 22.21 μm/1.42/g fibrous, fragmentary filmy	direct future cytotoxicity research to investigate any health implications associated with MNPs inhalation
Microplastics detected in cirrhotic liver tissue,2022	cirrhotic liver	Nile red staining, fluorescence microscopy, Raman spectroscopy	PVC, PET, PMMA, POM, PP	4–30 μm fragmentary microbeads	evaluate whether hepatic MNPs accumulation represents a potential cause in the pathogenesis of fibrosis or a consequence of cirrhosis and portal hypertension
First Evidence of Microplastics in Human Urine, a Preliminary Study of Intake in the Human Body,2022	urine	Raman spectroscopy	PVA, PVC, PP, PE	4–15 μm irregular	better characterize the level of risk and understand the possible transportation routes in biological fluids and tissues
Discovery and quantification of plastic particle pollution in human blood,2022	blood	Pyrolyzer-gas chromatography/mass spectrometry	PET, PP PS, PMMA	≥700 nm 1.6 μg/mL	evaluate whether blood exposure may affect immune regulation or have an immune basis susceptibility to disease
First evidence of microplastics in human placenta,2021	placenta	Raman spectroscopy	PP	5–10 μm spheres or irregular fragments	assess if the presence of MNPs in the human placenta may trigger immune responses or may lead to the release of toxic contaminants, resulting in harmful pregnancy
Raman Microspectroscopy Detection and Characterization of Microplastics in Human Breastmilk, 2022	breastmilk	Raman spectroscopy	PE, PVC, PP	1–12 μm spheres and irregular fragments	deepen the knowledge of the potential health impairment caused by MNPs internalization and accumulation, especially in infants, and to assess innovative, useful ways to reduce exposure to these contaminants during pregnancy and lactation

MNPs: microplastics and nanoplastics Yrs: publication years μFTIR: the micro Fourier transform interferometer.

PP: polypropylene PET: polyethylene terephthalate.

PVA: polyethylene vinyl acetate PVC: polyvinyl chloride PE: polyethylene.

PMMA: polymethyl methacrylate POM: polyoxymethylene PS: polymers of styrene.

## 2 Effects of MNPs on cellular characteristics

MNPs are major environmental pollutants with potential cellular effects and have raised concerns about their impact on human health. First, plastic particles can be categorized by size into microplastics (MPs) (5 mm–1 μm), submicroplastics (1 μm–100 nm), and nanoplastics (NPs) (less than 100 nm) (Caldwell et al., 2022), and their size is a key factor influencing their cellular interactions. Smaller particles, especially at the nanoscale, can more easily cross biological barriers and penetrate cells. A study has shown that MNPs smaller than 10–15 µm can be internalized by cells, leading to potentially cytotoxic effects (Prata et al., 2020). The ability of these particles to penetrate cell membranes and accumulate in cells raises great concern about their potential health effects. Second, MNPs come from primary and secondary sources. Primary MNPs are intentionally produced for use in products such as cosmetics and detergents, while secondary MNPs are the result of the degradation of larger pieces of plastic (Hanun et al., 2021). Plastics degrade in the environment due to UV radiation, wave action and temperature fluctuations and form secondary MNPs, which then enter various ecosystems and food chains. The surface properties of MNPs, such as roughness and charge, can significantly influence their interactions with biological systems. Particularly, weathering processes can change surface properties and make surfaces more hydrophobic or hydrophilic, which in turn influences the adsorption of environmental pollutants (Imhof et al., 2016). When MNPs enter the bloodstream, they interact with various proteins to form a protein corona. This aggregation can induce conformational changes and protein denaturation, resulting in the formation of non-biocompatible complexes. A more hydrophilic protein corona may mitigate the immune response and extend the circulation time of MNPs in the bloodstream, thereby influencing their biodistribution and potential toxicity (Gopinath et al., 2019). In addition, the type of polymer (such as polyethylene, polypropylene, polyvinyl chloride or polystyrene) can also influence the effects of MNPs on cells. Polystyrene MPs have been found to cause oxidative stress and neurotoxic effects in aquatic organisms (Danopoulos et al., 2022). However, the specific mechanisms by which MNPs cause cellular effects are not yet fully understood. Further research is needed to investigate these processes and develop strategies to mitigate the impact of MNPs on the environment and human health.

## 3 Observative findings between MNPs and CVD

Preclinical studies have shown that exposure to MNPs may be associated with the development and progression of CVD (Figure 1). In a recent prospective multicenter observational study, polyvinyl chloride was detected in more than 70 patients with asymptomatic carotid stenosis who had undergone carotid endarterectomy, and a cohort follow-up revealed that patients whose plaques contained MNPs had a greater composite risk of myocardial infarction, stroke, or death from all causes (Marfella et al., 2024). In another study, MPs were detected not only in carotid plaques but also in coronary arteries with atherosclerotic plaques and in aortas without plaques by pyrolysis gas chromatography/mass spectrometry (Py-GC/MS). Most importantly, the concentration of MPs in arteries, coronary arteries and carotid arteries containing atherosclerotic plaques is significantly greater than that in aortas without atherosclerotic plaques (Liu et al., 2024). These results indicate that MPs are involved in the development of atherosclerosis. In addition to the discovery of MPs in human organs potentially exposed to the external environment, recent studies using laser direct infrared chemical imaging systems and scanning electron microscopy have detected MPs in completely sealed human organ tissues (pericardial and extracardiac) from patients undergoing cardiac surgery. MPs have also been detected in membranes, myocardium and venous blood, providing direct evidence of MPs in patients undergoing cardiac surgery (Yang et al., 2023). Recent studies have highlighted that MNPs can induce alterations in cardiac structure and may even lead to damage in targer organs. In an angiotensin II-induced hypertension mouse model, the ingestion of a diet containing MNPs (specifically polystyrene) resulted in an increased cardiac hypertrophy index, reduced cardiac output, and increased renal fibrosis gene expression in mice (Fuckert et al., 2023). Furthermore, the ingestion of polystyrene MNPs was associated with increased body fat and weight gain in mice, thereby promoting the development of cardiometabolic disease phenotypes (Zhao et al., 2022). Another study demonstrated that MPs induced cardiac hypertrophy in both *in vivo* and *in vitro* models at low doses equivalent to human internal exposure (Zhou et al., 2023). Moreover, MPs have been shown to exert cardiotoxic effects, leading to pericardial edema, bradycardia (slow heart rate), abnormal rhythm, and myocardial fibrosis in fish cardiac tissues (Persiani et al., 2023). Additionally, one study indicated that in mouse models, oral administration of polystyrene NPs can induce myocardial structural abnormalities, elevate reactive oxygen species (ROS) levels in myocardial tissue, and trigger oxidative stress while activating the TGFβ1/Smad signaling pathway. This cascade results in increased fibrin and collagen deposition, ultimately contributing to myocardial fibrosis, which adversely affects both the structural integrity and functional performance of the heart (Lin et al., 2022). Similarly, polystyrene MPs induce cardiac fibrosis in rats by activating the Wnt/β-catenin signaling pathway and promoting cardiomyocyte apoptosis (Li et al., 2020). These studies suggest that the intake of MNPs may cause structural changes in the heart and even target organ damage, potentially representing an unrecognized risk factor for CVD.

## 4 Potential mechanism of the link between MNPs and CVD

**FIGURE 2:**
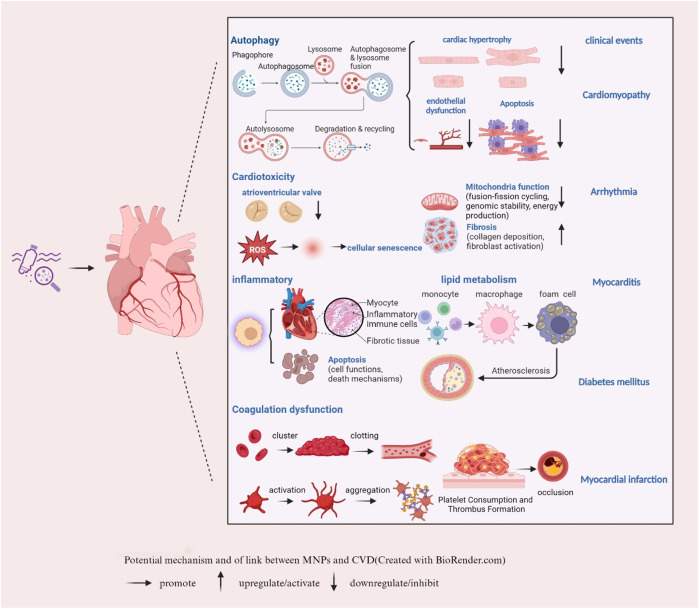
Potential mechanism of the link between MNPs and CVD (created with BioRender.com). → promote ↑ upregulate/activate ↓ downregulate/inhibit.

MNPs are closely related to CVD; therefore, the underlying mechanisms need to be further explored (Figure 2). MNPs can enter the human body in many ways, including drinking contaminated water, ingesting contaminated food (e.g., seafood), and inhaling MNPs in the air. Once in the body, they can enter the lungs and subsequently be transferred to the bloodstream, depending on size. One study has reported that the concentration of detectable plastic particles in human blood is approximately 1.6 μg/mL (Leslie et al., 2022). In comparison to conventional blood components, such as low-density lipoprotein (LDL), the concentration of plastic particles is typically low. However, with the rise in environmental pollution, it is anticipated that the concentration of plastic particles in the bloodstream may gradually increase over time. Thus, dietary habits and the consumption of biologically active nutrients are crucial for cardiovascular health (Cverenkarova et al., 2021).

### 4.1 Autophagy, MNPs and CVD

Autophagy is a highly conserved cellular process that plays a critical role in maintaining cellular homeostasis by recycling damaged organelles and proteins. This process is particularly important in the heart, where it helps to maintain the normal function of cardiomyocytes (heart muscle cells) and respond to various stressors, such as ischemia or pressure overload (Schiattarella and Hill, 2016). The potential disruption of autophagy by MNPs is of growing concern because it could have a significant impact on the progression of CVD. As Lu (2022) mentioned, there is evidence that exposure to NPs can interfere with autophagic processes within cells (Lu et al., 2022). The disruption of autophagy by MNPs could lead to the accumulation of damaged organelles and proteins in cardiomyocytes, impairing their function and potentially contributing to the development or progression of CVD (Chowdhury et al., 2023). Moreover, recent research has explored the impact of MPs on the vascular system. *In vitro* experiments were conducted on human umbilical vein endothelial cells (HUVECs) exposed to polystyrene MPs of varying sizes (0.5, 1, 5 μm) and concentrations. The findings revealed that 0.5 μm polystyrene MPs significantly reduced the activity, angiogenesis and migration ability of HUVECs while inducing autophagy and cell death (Lee et al., 2021). These results suggest that polystyrene MPs may impair endothelial cell function by disrupting angiogenesis signaling pathways and triggering autophagy/necrosis, indicating that they are major targets of circulating MPs. This highlights the importance of further research to understand the mechanisms by which MNPs affect autophagy and to assess the potential risks to human health.

### 4.2 Cardiotoxicity, MNPs and CVD

The triggering or acceleration of autophagy may be due to a cardiotoxic response from MPs (Zhou et al., 2023). Study has demonstrated that m6A modification may be implicated in MPs-induced cardiotoxicity by influencing the expression and function of non-coding RNAs (ncRNAs) (Zhang et al., 2023). Regarding the potential effects of environmental contamination with NPs on human induced pluripotent stem cells during early human development, gene enrichment analysis revealed that the development of the atrioventricular valve and the dysfunction of cellular components, including the extracellular matrix, are significantly impaired (Bojic et al., 2020). In addition, polystyrene-induced cardiotoxicity in a rat model increased the serum levels of troponin I and creatine kinase MB (CK-MB), leading to myocardial fibrosis via activation of the Wnt/β-catenin pathway (Li et al., 2020). Another study showed that MPs may exacerbate cardiac structure and function by damaging mitochondrial integrity, causing cardiomyocyte apoptosis and pyroptosis (Wei et al., 2021). In addition, one study suggested that MPs accelerate premature vascular aging through the CDK5 signaling pathway, which is mediated by reactive oxygen species (Wang K. et al., 2023). These studies suggest that the cardiotoxicity of MNPs may cause cardiac dysfunction through mechanisms such as oxidative stress, mitochondrial dysfunction, and inflammation. Although plastic particles smaller than MPs, such as NPs, are thought to play an important role in human body toxicity, studies focusing on their cardiovascular effects are scarce. Recently, a study reported that exposure to polystyrene NPs with varying functional groups significantly exacerbates type 2 diabetes mellitus (T2DM)-like conditions primarily through inhibition of the P-AKT/P-FoxO1 signaling pathway (Wang et al., 2024). This study underscores the potential of NPs pollution to contribute to the development and progression of cardiometabolic disorders. Notably, some researchers have recently investigated the combined cardiotoxic effects of co-exposure to polystyrene NPs and cadmium (Cd) on mice. One study revealed that simultaneous exposure to polystyrene NPs and Cd resulted in the upregulation of gene and protein expression associated with pyroptosis, apoptosis and necrosis, which in turn led to growth restriction and damage to the myocardial microstructure in mice (Ye et al., 2024). This study provides insights into the molecular mechanism of myocardial damage induced by environmental pollutants and provides a scientific basis for further research into the combined toxicological effects of polystyrene NPs and heavy metals. In summary, the growing evidence of the cardiotoxicity of MNPs leading to CVD highlights the necessity for further investigation of the health risks associated with the accumulation of plastic waste in the environment.

### 4.3 Inflammatory response, lipid metabolism, MNPs and CVD

Inflammatory responses and lipid metabolism play important roles in the occurrence and development of CVD. Recent studies have indicated the impact of MNPs on the systemic immune system, with a focus on the effects on the cardiovascular system (Prado et al., 2023). Studies have suggested that polystyrene MPs induce cardiotoxicity in chickens through the ROS-driven NF-κB-NLRP3-GSDMD and AMPK-PGC-1α axes, triggering oxidative stress, myocardial apoptosis, inflammation, and disruption of mitochondrial and energy metabolism (Zhang et al., 2022). *In vitro* experiments have shown that MNPs can increase the inflammatory response by activating the inflammasome NLRP3, which has been confirmed in THP-1 cells (macrophages) (Busch et al., 2022). Additionally, NPs were found to enhance the release of proinflammatory cytokines in human monocytes and monocyte-derived dendritic cells (Weber et al., 2022). Moreover, polystyrene NPs may also contribute to the development of atherosclerosis by affecting mitochondria-mediated oxidative stress, promoting foam cell differentiation and altering lipid metabolism (Florance et al., 2022; Florance et al., 2021). Furthermore, a study showed that exposure to polystyrene MPs can disturb lipid metabolism and increase oxidative stress and inflammation in the cardiovascular system of rats, possibly contributing to cardiac metabolic diseases (Song et al., 2024). The mechanisms involved are strikingly similar to those observed with NPs. Oxidized low-density lipoprotein (Ox-LDL), with a diameter of approximately 20–30 nm, plays a crucial role in the occurrence and progression of atherosclerosis. It not only promotes the formation of foam cells but also triggers inflammation and affects endothelial cell function (Poznyak et al., 2020). One study indicated that co-exposure to polystyrene NPs with a diameter of 100 nm and Ox-LDL significantly induces lipid accumulation in macrophages, as well as oxidative stress and inflammatory responses (Wen et al., 2024). Furthermore, another study suggested that 50 nm polystyrene NPs and long-chain acylcarnitine (LCAC) alone exacerbate lipid accumulation by upregulating macrophage receptors (MARCO) in Ox-LDL-activated foam cells, thereby promoting the development of atherosclerosis (Wang B. et al., 2023). These findings provide new insights into the mechanisms linking MNPs and atherosclerosis. More recently, bisphenol A (BPA), a typical leachable additive from MPs and one of the most prolific bulk chemicals, has been shown to be widely distributed in sediments, sewage and wastewater treatment plants (Fu et al., 2024). Exposure to BPA and its analogs, bisphenol S (BPS) and bisphenol F (BPF), leads to the accumulation of genomic instability in human peripheral blood cultures by increasing micronuclei frequencies and altering the expression of human endogenous retrovirus (HERV) *env* genes (Ruberto et al., 2022). These results prove that MNPs can cause inflammatory reactions, leading to vascular endothelial dysfunction by activating inflammatory cells, inducing genomic instability and releasing inflammatory mediators, such as cytokines and oxidative stress substances. They can also affect lipid metabolism and promote the development of atherosclerosis.

### 4.4 Coagulation system, MNPs and CVD

Since studies have reported that MNPs, especially MPs, are found in human blood clots and can accumulate in arteries, scientists believe that the risk of MPs exposure is seriously underestimated. In terms of the coagulation system, the study also revealed a significant positive correlation between the number of particles in the thrombus and the platelet count after adjusting for potential confounding factors (Wu et al., 2023). Moreover, one study reported that the interaction between MNPs, thrombosis and the cardiovascular system is highly dependent on their size and surface chemistry, suggesting that the effect of MPs is closely related to their size and surface chemistry. In addition, surface-charged particles (such as aminated MNPs) may promote platelet aggregation and thrombosis by interacting with receptors on the surface of platelets (Lett et al., 2021). A similar study conducted in a rat venous thrombosis model showed that intravenous injection of polystyrene NPs enhanced thrombosis, possibly because amine-modified polystyrene triggers prothrombotic activation of red blood cells, leading to thrombosis (Kim et al., 2022). These findings help to elucidate how MNPs affect the coagulation system and lead to CVD.

## 5 Potential drug targets for MNPs-related CVD

**FIGURE 3:**
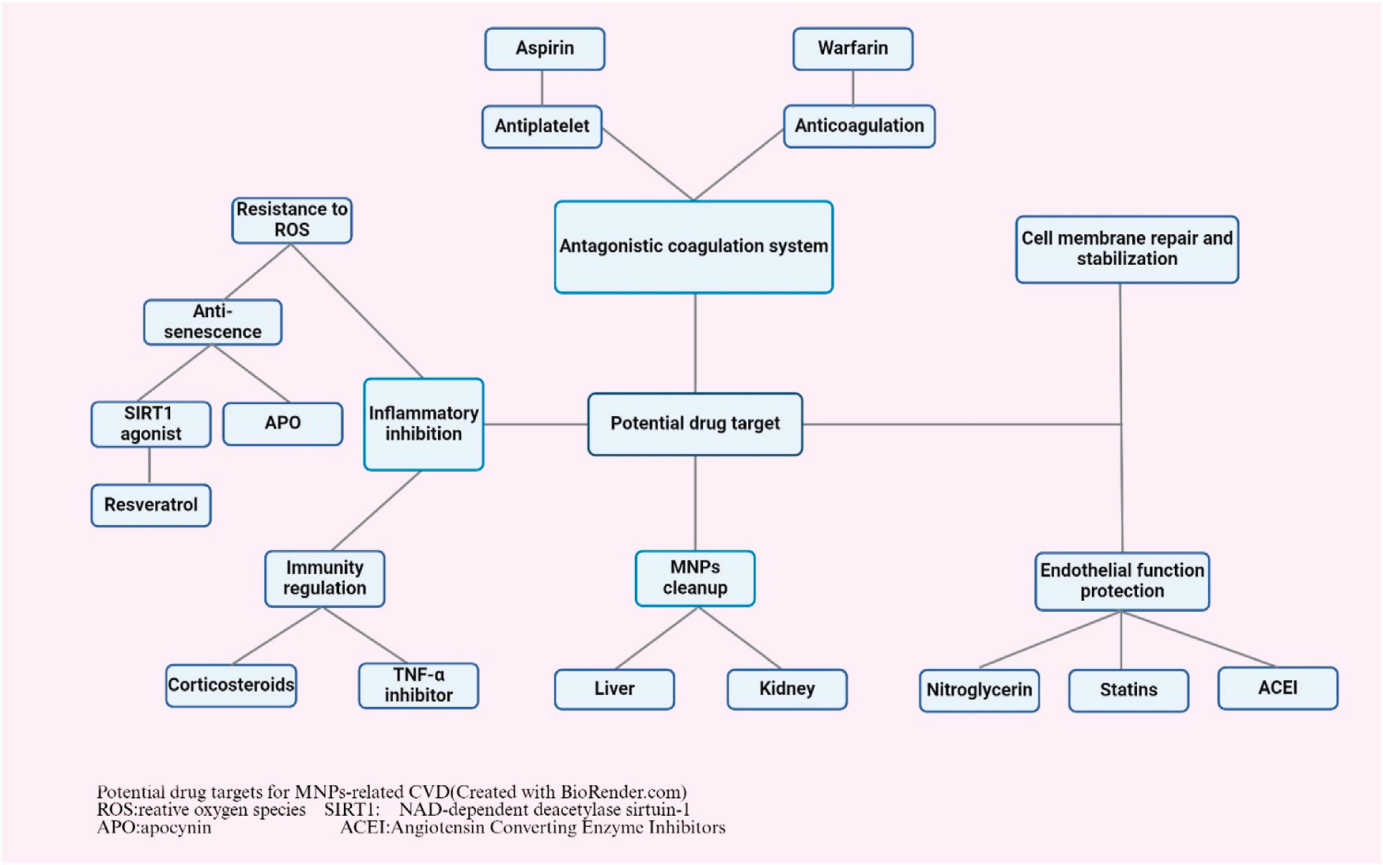
Potential drug targets for MNPs-related CVD (created with BioRender.com). ROS: relative oxygen species; SIRT1: NAD-dependent deacetylase sirtuin-1; APO: apocynin; ACEI: Angiotensin converting enzyme inhibitors.

Although current research on potential drug treatment targets for CVD is still in its infancy, some potential treatment targets can be speculated based on the various mechanisms by which MNPs affect the cardiovascular system (Figure 3). Due to their small size and large surface area, MNPs can interact with cells and tissues in organisms and induce local or systemic inflammatory responses. A recent study suggested that MPs significantly aggravate systemic inflammation caused by a high-fat diet in individuals with obesity by activating peripheral and central inflammatory immune cells (Lee et al., 2023). These inflammatory responses may be associated with a range of CVDs. Anti-inflammatory treatments typically involve the use of drugs to inhibit inflammatory pathways, reduce the production of inflammatory mediators, and modulate the immune response. Moreover, the oxidative stress cascade was activated by exposure to MNPs. A study on the effects and potential mechanisms of polystyrene NPs on the aging of porcine coronary artery endothelial cells showed that exposure to polystyrene NPs promotes premature aging of endothelial cells, as evidenced by the loss of cell proliferation, activation of reactive oxygen species and impaired vasodilation, whereas antioxidants (N-acetylcysteine, NAC), NADPH oxidase inhibitors (apocynin, APO) and Sirt1 activators (resveratrol, RES) had a preventive effect on polystyrene-induced endothelial cell senescence and dysfunction (Shiwakoti et al., 2022). Therefore, anti-inflammatory and antioxidant treatments may help to reduce the inflammatory damage caused by MNPs, protect tissues from damage, and reduce the negative effects of MNPs on the cardiovascular system and other organs (Hu and Palic, 2020). Anti-inflammatory drugs are a potential treatment option. Nonsteroidal anti-inflammatory drugs (NSAIDs), such as aspirin, corticosteroids, and biologics, may reduce the risk of MNPs by inhibiting inflammatory factors such as NF-κB, TNF-α, IL-1β, IL-6, and IL-8 (Henein et al., 2022). Treatment with antioxidants may help to mitigate this potential effect. In one study, melatonin was found to reduce ischemia‒reperfusion injury caused by MPs accumulation, suggesting that natural antioxidants may help protect cells from oxidative damage (Missawi et al., 2023). MNPs may also affect the coagulation system. One study reported that NPs can be detected in human blood and that exposure to NPs can lead to coagulation disorders and prothrombotic conditions (Wang X. et al., 2023). Therefore, medications such as antiplatelet agents (aspirin), anticoagulants (warfarin) and fibrinolytics may help prevent or treat thrombosis caused by MNPs. In addition, MNPs may impair endothelial function, thereby promoting leukocyte adhesion, a critical step in the processes of vascular inflammation and the development of atherosclerosis (Vlacil et al., 2021). Therefore, drugs to protect endothelial cells, such as nitrates, statins and angiotensin converting enzyme inhibitors (ACEIs), may help maintain vascular health. Similarly, exposure to MPs is mainly associated with changes in the physicochemical or biological properties of red blood cells, including a reduction in membrane composition, disruption of bilayer thickness and intrinsic lipid curvature. These results indicate that the membrane function of blood cells is impaired (Wang et al., 2022). Therefore, treatments that promote the repair and stabilization of cell membranes may mitigate the harmful effects of MPs. The last and most fundamental treatment possible is the removal of MNPs. Although there are currently no drugs that specifically target MNPs, researchers could explore ways to promote the elimination of MNPs from the body, such as by enhancing the detoxification functions of the liver and kidneys and their excretion via the gastrointestinal tract. In summary, the investigation of drug treatment targets for CVD caused by MNPs is an emerging field of research that requires further scientific research.

## 6 Conclusions and future perspectives

In summary, the evidence linking MNPs to CVD is rapidly emerging, highlighting critical pathways through which these environmental pollutants may contribute to cardiovascular health risks. Current studies demonstrate that MNPs can infiltrate human tissues and trigger a range of toxicological responses, including inflammation, oxidative stress, and alterations in lipid metabolism, which collectively may exacerbate CVD. Although significant strides have been made in understanding the cellular and molecular mechanisms involved, extensive research is still needed to elucidate the precise pathways through which MNPs impact cardiovascular health. Future studies should prioritize large-scale epidemiological investigations and long-term cohort studies to provide comprehensive insights into the correlation between MNPs exposure and CVD outcomes. Furthermore, there is an urgent need for the development of innovative strategies aimed at reducing MNPs pollution, which could mitigate its potential health impacts. This may include advancing recycling technologies, promoting biodegradable alternatives, and improving waste management practices. Ultimately, a multidisciplinary approach, combining toxicological research with public health policy and environmental science, will be essential to address the challenges posed by MNPs and safeguard cardiovascular health in the context of an increasingly plastic-laden environment.
